# Pandemic clone USA300 in a Brazilian hospital: detection of an emergent lineage among methicillin-resistant *Staphylococcus aureus* isolates from bloodstream infections

**DOI:** 10.1186/s13756-022-01154-3

**Published:** 2022-09-14

**Authors:** Mariana Fernandes Augusto, Débora Cristina da Silva Fernandes, Tamara Lopes Rocha de Oliveira, Fernanda Sampaio Cavalcante, Raiane Cardoso Chamon, Adriana Lúcia Pires Ferreira, Simone Aranha Nouér, Infection Control Group HUCFF/UFRJ, Ana Pereira Rangel, Anna Carla Castiñeiras, Christiany Moçali Gonçalez, Joana Freire, Luiz Felipe Guimarães, Raquel Batista, Kátia Regina Netto dos Santos

**Affiliations:** 1 Laboratório de Infecção Hospitalar, Departamento de Microbiologia Médica, Instituto de Microbiologia Paulo de Góes, Universidade Federal do Rio de Janeiro, CCS, Bloco I, Sala I2-010 - Cidade Universitária, Rio de Janeiro, RJ CEP 21941-590 Brazil; 2 Departamento de Microbiologia, Imunologia e Parasitologia, Faculdade de Ciências Médicas, Universidade do Estado do Rio de Janeiro, Rio de Janeiro, Brazil; 3 Centro Multidisciplinar de Macaé, Universidade Federal do Rio de Janeiro, Macaé, Brazil; 4 Departamento de Patologia, Faculdade de Medicina, Universidade Federal Fluminense, Niterói, Rio de Janeiro Brazil; 5 Serviço de Patologia Clínica, Hospital Universitário Clementino Fraga Filho, Universidade Federal do Rio de Janeiro, Rio de Janeiro, Brazil; 6 Faculdade de Medicina, Hospital Universitário Clementino Fraga Filho, Universidade Federal do Rio de Janeiro, Rio de Janeiro, Brazil

**Keywords:** *S. aureus*, Bloodstream infections, MRSA, USA300, USA100, PVL genes

## Abstract

**Background:**

*Staphylococcus aureus* is one of the leading causes of bloodstream infections (BSI) worldwide. In Brazil, the hospital-acquired methicillin-resistant *S. aureus* USA100/SCC*mec*II lineage replaced the previously well-established clones. However, the emergence of community-associated (CA) MRSA lineages among hospitalized patients is an increasing issue.

**Methods:**

Consecutive *S. aureus* isolates recovered from BSI episodes of patients admitted between January 2016 and December 2018 in a Brazilian teaching hospital were tested for antimicrobial resistance, their genotypic features were characterized, and the clinical characteristics of the patients were evaluated.

**Results:**

A total of 123 *S. aureus* isolates were recovered from 113 patients. All isolates were susceptible to linezolid, teicoplanin and vancomycin and 13.8% were not susceptible to daptomycin. Vancomycin MIC_50_ and MIC_90_ of 2 mg/L were found for both MRSA and MSSA isolates. The MRSA isolation rate was 30.1% (37/123), and 51.4% of them carried the SCC*mec* type II, followed by SCC*mec*IV (40.5%). Among the 37 MRSA isolates, the main lineages found were USA100/SCC*mec*II/ST5 and ST105 (53.7%) and USA800/ST5/SCC*mec*IV (18.9%). Surprisingly, six (16%) CA-MRSA isolates, belonging to USA300/ST8/SCC*mec*IVa that carried PVL genes and the ACME cassette type I, were detected. These six patients with USA300 BSI had severe comorbidities, including cancer, and most had a Charlson score ≥ 5; furthermore, they were in wards attended by the same health professionals. MRSA isolates were associated with hospital acquired infections (*p* = 0.02) in more elderly patients (*p* = 0.03) and those diagnosed with hematologic cancer (*p* = 0.04). Among patients diagnosed with MRSA BSI, 19 (54.3%) died.

**Conclusions:**

The pandemic MRSA USA300 was detected for the first time in the Brazilian teaching hospital under study, and its cross-transmission most probably occurred between patients with BSI. This lineage may already be circulating among other Brazilian hospitals, which highlights the importance of carrying out surveillance programs to fight multidrug resistant and hypervirulent isolates.

## Background

S*taphylococcus aureus* is one of the most common causes of bloodstream infections (BSI) worldwide [1, 2]. The presence of an indwelling device or a skin and soft tissue infection (SSTI) associated to hospital (HA) or community (CA) BSI are the most common sources of this pathogen [3]. *S. aureus* BSIs, especially those caused by MRSA, cause higher rates of morbidity and mortality and the costs associated to such infections are burdensome, especially in middle and low-income countries [4]. In addition, the presence of methicillin-resistant *S. aureus* (MRSA) as the cause of BSI represents a therapeutic challenge in health care institutions [5]. However, there has been a reduction in the number of MRSA isolated from BSI worldwide [1, 6]. Diekema and coworkers [1] showed that *S. aureus* was the main Gram-positive bacteria isolated from BSI in a 20-year surveillance period, between 1997 and 2016, involving 45 nations, including those in Latin America. The authors showed that there was a decline in the number of MRSA isolates over these years and that daptomycin resistance among *S. aureus* isolates remained rare (< 0.1%); furthermore, during that period there was no trend toward an increase in the vancomycin MIC.

Over recent years, the substitution of well-established MRSA lineages has been described in hospital environments [7, 8]. In Brazilian hospitals, the previously predominant MRSA lineage, known as the Brazilian Endemic Clone (BEC/ST239) has been fully replaced by the New York/Japan lineage among BSI isolates (USA100/ST5 or ST105) [9–11]. Lately, our group also detected the emergence of USA1100/ST30, a CA-MRSA lineage in Brazilian hospital settings [8, 10]. Curiously, in Brazil, the pandemic clone USA300/ST8/SCC*mec*IVa and the USA300-Latin American Variant (LV)/SCC*mec*IVc-e lineages have remained rare [11]. These related lineages present different SCC*mec* subtypes and the USA300-LV lacks the operon ACME [12]. However, these lineages could carry the Panton-Valentine leukocidin genes and are considered hypervirulent, since once they are present in the environment; they can spread easily, leading to infections with high mortality rates [13].

Molecular studies have helped the epidemiological surveillance of *S. aureus* BSI, highlighting the constant clonal change that has been taking place in health institutions and the dissemination of MRSA clones that can lead to higher rates of morbidity and mortality [14]. Moreover, constant monitoring of *S. aureus* BSI can also help surveillance programs in containing the spread of their resistance and virulence. In recent years, our group conducted a surveillance of *S. aureus* BSI in a University Hospital of Rio de Janeiro, Brazil, to identify the emergence of new clones or new resistance traits that may alter the epidemiology and treatment of these infections. This study aims to continue this surveillance through the characterization of *S. aureus* isolates from BSI over a three-year period in relation to antimicrobial resistance and virulence associated with different clones and correlate these results with clinical data of the patients.

## Methods

### Clinical isolates and setting

This prospective cohort study evaluated the phenotypic and molecular profiles of *S. aureus* isolates recovered from consecutive episodes of BSI occurring in adult patients (> 18 years old), who were admitted to the Hospital Universitário Clementino Fraga Filho (HUCFF) between January 2016 and December 2018. HUCFF is a tertiary public teaching hospital in Rio de Janeiro, Brazil, which currently has 300 active beds.

The first isolate of an episode of *S. aureus* BSI were analyzed, with subsequent documentation of blood cultures, clinical improvement, and anti-staphylococcal therapy. Demographic data, classification of the BSI episode [15], treatment and length of stay were collected from each patient.

All blood cultures were processed using the BacT/ALERT system (BioMerieux, Durham, NC, USA). Bacterial identification was performed by the automated VITEK2® system (BioMerieux) and confirmed by MALDI-TOF MS (Matrix Assisted Laser Desorption Ionization/ Time of Flight Mass Spectrometry) (Bruker Daltonics, Billerica, MA, USA) [16].

### Antimicrobial susceptibility tests and SCC*mec* typing

Susceptibility to methicillin and trimethoprim-sulfamethoxazole (TMP/STX) was determined using the disk-diffusion test method (Oxoid, Cambrigde, UK) according to the CLSI guidelines [17]. Minimum inhibitory concentrations (MICs**)** for daptomycin, linezolid, oxacillin, teicoplanin and vancomycin (Sigma-Aldrich Chemical Company, St Louis, MO, USA) were determined by the broth microdilution (BMD) method, using fresh cation-adjusted Muller-Hinton broth (CAMHB). Daptomycin received a supplement of calcium (50 μg/mL) [17]. The ceftaroline MICs were determined by the gradient diffusion method (Etest®, BioMérieux) for all MRSA isolates. *S. aureus* ATCC 25923 and ATCC 29213 were used as controls for the disk-diffusion and BMD tests, respectively.

The *SCCmec* typing [18] and subtyping [19] for isolates resistant to oxacillin by the disk-diffusion method were assessed by multiplex polymerase chain reaction (PCR), using bacterial DNA obtained using the QIAamp DNA Mini Kit (QIAGEN, Hilden, Germany). The *Staphylococcus* strains used as positive controls are described in Salgueiro et al. [20].

### Phenotypic tests for screening of hVISA isolates

All isolates presenting a MIC ≥ 2 mg/L for vancomycin were screened for the hVISA (heteroresistant vancomycin-intermediate *S. aureus*) phenotype [21, 22]. Plates with BHI agar (Becton Dickson, Franklin Lakes, NJ, USA), containing 3 and 4 mg/L of vancomycin were swabbed with 0.5 McFarland standard suspension (10^8^ CFU/mL) of *S. aureus* and incubated at 35 to 37 °C for 24 h. Reduced susceptibility to vancomycin was defined as the growth of one or more colony-forming units (CFU) at either of the two concentrations. BHI agar plates with 4 mg/L of vancomycin and supplemented with 16 g/L of pancreatic casein digestion (Merck), were also inoculated with four 10μL spots from a 0.5 McFarland inoculum [22]. An isolate was considered to have reduced susceptibility to vancomycin if at least one spot had two or more CFUs. *S. aureus* ATCC 29,213 (MSSA) and Mu3 (hVISA) were used as controls [23].

### Genotypic profile of MRSA isolates

All MRSA isolates were typed by pulsed-field gel electrophoresis (PFGE) after digestion of whole cell DNA with *Sma*I in a CHEF-DRIII system (Bio-Rad, Richmond, CA, USA), as previously described [24]. The PFGE fingerprints were compared by the unweighted pair-group method with arithmetic mean clustering analysis, applying the Dice correlation coefficient. Isolates with four or fewer bands of difference or minimum of 80% similarity were classified as belonging to the same genotype [25]. The clonal lineages were defined by comparison with national [10] and international clones [26]. Representative isolates from each genotype, identified in PFGE, underwent Multilocus Sequence Typing (MLST) [27].

### Detection of virulence genes

The detection of PVL genes was carried out in all MRSA isolates using PCR [28]. Isolates belonging to the USA300/ST8/SCC*mec*IV lineage also underwent ACME typing [29]. The *Staphylococcus* strains used as positive controls are described in Schuenck et al. [28].

### Statistical analysis

Categorical variables were compared using the chi-square or Fisher exact tests, and continuous variables were compared using the Wilcoxon test. A *p* value < 0.05 was considered to be statistically significant. All analyses were performed using SPSS 21.0 for Windows (SPSS, Inc).

## Results

### Baseline characteristics of patients presenting *S. aureus* BSI

During the study period, 113 patients developed *S. aureus* BSI. Ten of them presented two BSI episodes. Thus, a total of 123 *S. aureus* isolates were recovered. Most of the patients (53.1%; 60/113), who were men, presented at least one comorbidity (89.4%) and developed central line-associated BSI (55.8%) or secondary bacteremia due to SSTI (24.8%). MRSA was associated to more elderly patients (*p* = 0.03), hematologic cancer (*p* = 0.04) and hospital-associated BSI (*p* = 0.02). A total of 50 (44.2%) patients died, and among those diagnosed with MRSA BSI, 54.3% died (Table 1).

**Table 1:** Baseline characteristics of 113 patients presenting methicillin-resistant *Staphylococcus aureus* bloodstream infections

Clinical characteristics	MSSA (N = 78)	MRSA (N = 35)	TOTAL (N = 113)	*p* value
Gender, n (%) = male	41 (52.6)	19 (54.3)	60 (53.1)	1.00
Age (years), mean (range)	58.4 (18–98)	64.5 (39–87)	62 (18–98)	**0.03**
*Underlying comorbidities, n (%)*				
Diabetes	22 (28.2)	11 (31.4)	33 (29.2)	0.83
Previous neutropenia	2 (2.6)	3 (8.6)	5 (4.4)	0.33
Acute renal insufficiency	3 (3.8)	3 (8.6)	6 (5.3)	0.38
End stage renal disease	35 (44.9)	8 (22.9)	43 (38.0)	0.16
Cardiopathy	56 (71.8)	21 (60.0)	77 (68.1)	0.62
Pneumopathy	5 (6.4)	7 (20.0)	12 (10.6)	0.10
Neurologic disease	7 (8.9)	2 (5.7)	9 (8.0)	0.72
Hepatopathy	6 (7.7)	6 (17.1)	12 (10.6)	0.20
Renal replacement therapy	30 (38.5)	8 (22.9)	38 (33.6)	0.30
Solid cancer	12 (15.4)	7 (20.0)	19 (16.8)	0.60
Hematologic cancer	1 (1.3)	4 (11.4)	5 (4.4)	**0.04**
Autoimmune disease	1 (1.3)	3 (8.6)	4 (3.5)	0.09
Solid organ transplant	11 (14.1)	3 (8.6)	14 (12.4)	0.55
Previous BSI	11 (14.1)	7 (20.0)	18 (15.9)	0.59
*Acquisition, n (%)*				
HA-BSI	35 (44.9)	26 (74.6)	61 (53.9)	**0.02**
CA-BSI	22 (28.2)	5 (14.3)	27 (23.9)	
HCA-BSI	21(26.9)	4 (11.4)	25 (22.1)	
*Infection source, n (%)*				
Central line vascular catheter	42 (53.8)	21 (60.0)	63 (55.8)	0.5
Pulmonary	5 (6.4)	1 (2.9)	6 (5.3)	
SSTI	18 (23.1)	10 (28.6)	28 (24.8)	
Primary bacteremia	13 (16.7)	3 (8.6)	16 (14.2)	
*Outcome, n (%)*				
Death	31 (39.7)	19 (54.3)	50 (44.2)	0.15

*p* values in bold were considered statistically significant

MSSA, Methicillin-susceptible *Staphylococcus aureus;* MRSA, Methicillin-resistant *Staphylococcus aureus;* BSI, Bloodstream infection; HA, Hospital-acquired; CA, Community-acquired; HCA, Healthcare-associated; SSTI, Skin and soft tissue infection, ND, Not determined; A value of *p* ≤ 0.05 was considered statistically significant; Only the first episode was considered for patients that presented two BSI episodes

### Antimicrobial susceptibility, hVISA screening and SCC*mec* typing

According to the disk-diffusion method, 30.1% (37/123) of *S. aureus* isolates were resistant to cefoxitin and were characterized as MRSA. Overall, 51.4% (19/37) of these isolates carried the SCC*mec*II lineage, followed by SCC*mec*IV (15/37; 40.5%), SCC*mec*III (2/37; 5.4%) and SCC*mec*V (1/37; 2.7%). Among the SCC*mec*IV isolates those that were subtyped as SCC*mec*IVa belonged to USA300 lineage. Only the SCC*mec*III isolates were resistant to TMP/STX.

Table 2 shows the MIC results. Both MRSA and MSSA isolates presented MIC_50_ and MIC_90_ = 2 mg/L for vancomycin. This MIC value was found in 90% of the SCC*mec*II isolates and in 53.3% of those that carried the SCC*mec*IV. The ceftaroline MICs in the MRSA isolates ranged from 0.25 to 0.75 mg/L. The oxacillin MICs, MIC_50_ and MIC_90_, were 256 and ≥ 256 mg/L, respectively in the MRSA isolates, and 1 and 2 mg/L among the MSSA isolates. All isolates were susceptible to linezolid, teicoplanin and vancomycin. Seventeen (13.8%) *S. aureus* isolates were non-susceptible to daptomycin, and among them, five (29.4%) were MRSA isolates.

**Table 2:** Minimal inhibitory concentration of different antimicrobials against 123 *Staphylococcus aureus* isolates from bloodstream infections

Antimicrobial	MSSA (N = 86)				MRSA (N = 37)			
	MIC range	MIC_50_	MIC_90_	Non-susceptible^a^ isolates (%)	MIC range	MIC_50_	MIC_90_	Non-susceptible^a^ isolates (%)
Oxacillin	0.25–2	1	2	0	16 to ≥ 256	256	≥ 256	100^b^
Vancomycin	0.5–2	2	2	0	0.5–2	2	2	0
Teicoplanin	0.25–2	0.5	1	0	0.25–1	0.5	0.5	0
Linezolid	0.25–1	1	1	0	0.25–2	0.5	1	0
Daptomycin	0.5–2	1	2	13.9	0.5–2	1	2	13.5
Ceftaroline	NA	NA	NA	NA	0.25–0.75	0.5	0.75	0

Minimal inhibitory concentration (MIC) values for oxacillin, vancomycin, teicoplanin, linezolid and daptomycin were determined by the broth microdilution method and for ceftaroline by the E-test® and presented in mg/L

MSSA, Methicillin-susceptible *Staphylococcus aureus*; MRSA, Methicillin-resistant *Staphylococcus aureus*; NA, Not applicable

^a^Determined according to CLSI interpretation criteria

^b^Including oxacillin-resistant isolates

All the 72 (58.5%) isolates that presented a vancomycin MIC = 2 mg/L were screened for the hVISA phenotype. However, no hVISA phenotype was found.

### Genotypic profiles and virulence genes in MRSA isolates

**Fig. 1:**
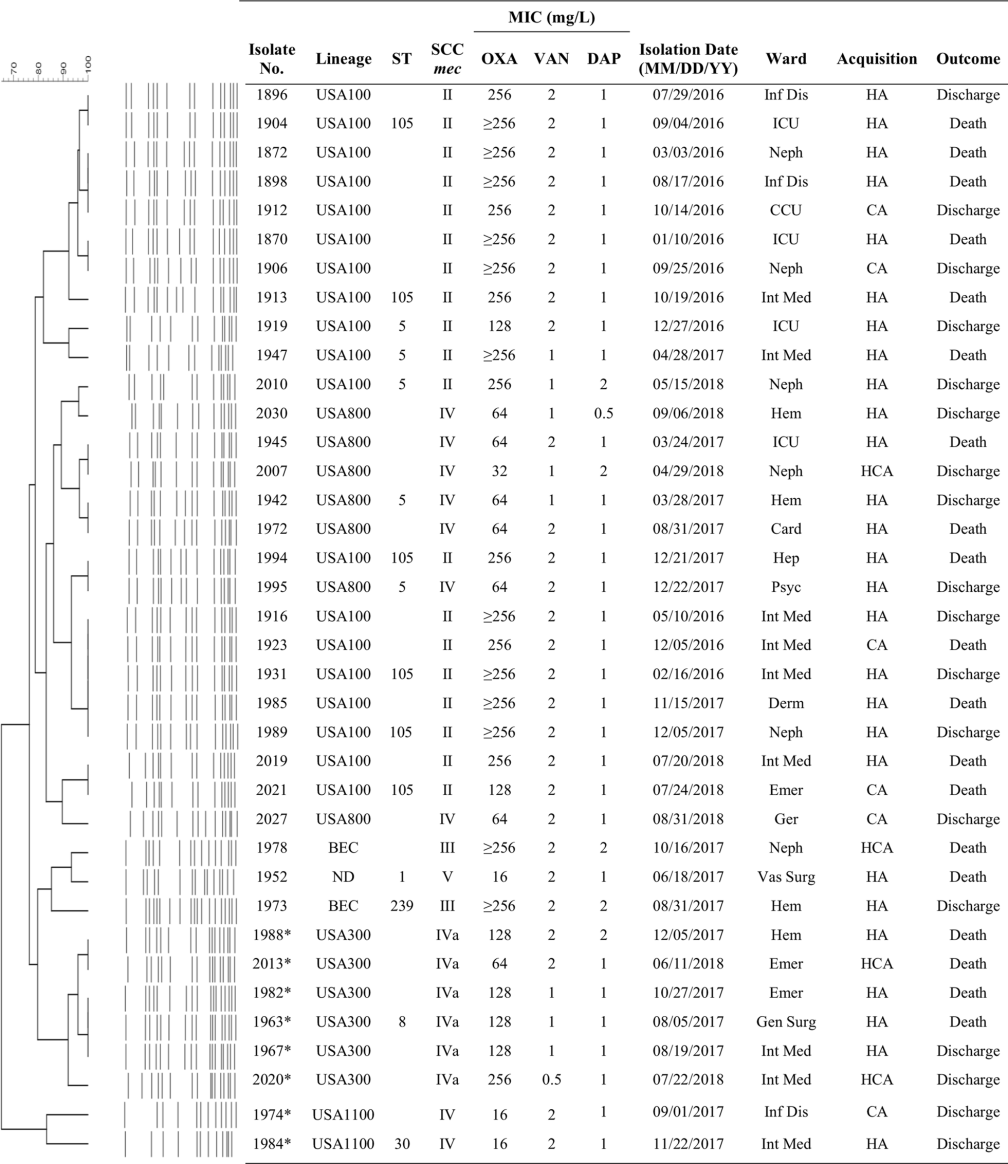
Dendrogram of the PFGE patterns and characteristics related to the genetic background of 37 MRSA isolates recovered from bloodstream infections. Isolates showing a similarity coefficient ≥ 80% were considered genetically related. *PVL genes positive isolates; ST, Sequence type; SCC*mec*, Staphylococcal cassette chromosome *mec*; MIC, Minimum inhibitory concentration; OXA, oxacillin; VAN, vancomycin; DAP, daptomycin; MM, Month; DD, Day; YY, Year; Hem, Hematology; Neph, Nephrology; Vas Surg, Vascular Surgery; Emer, Emergence; Gen Surg, General Surgery; Int Med, Internal Medicine; Hep, Hepatology; Psyc, Psychiatry; Derm, Dermatology; ICU, Intensive Care Unit; Card, Cardiology; Ger, Geriatrics; Inf Dis, Infectious Disease; CCU, Coronary Care Unit; HA, Hospital-associated; HCA, Healthcare-associated; CA, Community-associated; BEC, Brazilian endemic clone; ND, not determined

The PFGE patterns and general characteristics of 37 MRSA isolates recovered from BSI are presented in Fig. 1. The isolates were clustered among specific lineages: USA100/SCC*mec*II of ST5 (3 isolates; 8.1%) and ST105 (16; 43.2%), a single-locus variant (SLV) of ST5; USA800/ST5/SCC*mec*IV (7; 18.9%); USA300/ST8/SCC*mec*IVa (6; 16.2%); USA1100/ST30/SCC*mec*IV (2; 5.4%) and BEC/ST239/SCC*mec*III (2; 5.4%). One isolate carrying the *SCCmec* type V lineage and related to ST1 was also found. The PVL genes were found in all isolates of the USA300/ST8 and USA1100/ST30 lineages.

### Characteristics of the USA300 isolates

**Fig. 2:**
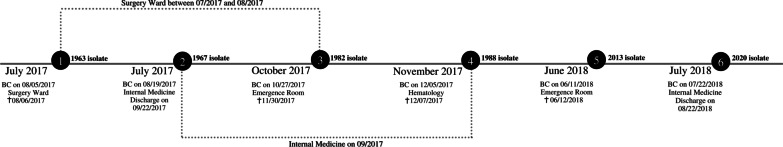
Timeline distribution of the six patients who presented bloodstream infections caused by USA300/ST8/SCC*m*ecIVa isolates according to hospital ward. BC—Blood culture positive for MRSA USA300; †Death as outcome

All the USA300/ST8/SCC*mec*IVa isolates (1963, 1967, 1982, 1988, 2013, and 2020) carried both PVL virulence genes and ACME cassette type I (Table 3). These isolates were recovered from different patients, who had developed hospital or healthcare-associated BSIs. All these individuals had severe comorbidities and all, but one patient (isolate number 2020), had a central line in situ when diagnosed with BSI. Four patients presented a Charlson score ≥ 5. Most of these patients were undergoing cancer treatment, although not at the same unit. Figure 2 shows the timeline distribution of the six patients with BSI caused by the USA300 according to their hospital ward. A direct nosocomial transmission could not be clearly established, although some patients were admitted to the hospital only a few days apart, and there was a ≥ 90% similarity between the isolates (Fig. 1). However, the wards occupied by these patients are physically close to each other (along the same corridor), and the same healthcare workers and cleaning staff attended these wards.

**Table 3:** Clinical and microbiological characteristics related to six patients that presented bloodstream infections caused by the pandemic MRSA USA300/ST8/SCC*mec*IVa lineage

Isolate No	Admission (MM/DD/YY)	Gender/Age (Y)	Comorbidities	Charlson score	Ward	Acquisition	Antimicrobial treatment	Isolation date (MM/DD/YY)	MIC (mg/L)			Virulence	PFGE Pulsotype
									OXA	VAN	DAP		
1963†	07/11/17	M/79	SC	5	Gen Surg	HA	VAN	08/05/17	128	1	1	PVL, ACME-I	A1
1967	07/21/17	M/43	SC	2	Int Med	HA	VAN	08/19/17	128	1	1	PVL, ACME-I	A1
1982†	10/26/17	M/68	D, C, SC	10	Emer	HA	VAN	10/27/17	128	1	1	PVL, ACME-I	A1
1988†	11/06/17	M/61	HC	6	Hem	HA	PIP + TAZ	12/05/17	128	2	2	PVL, ACME-I	A2
2013†	06/11/18	F/40	L, ESRD, C, P	3	Emer	HCA	VAN	06/11/18	64	2	1	PVL, ACME-I	A2
2020	07/20/18	M/67	D, C, HC	5	Int Med	HCA	VAN + CEFP	07/22/18	256	0.5	1	PVL, ACME-I	A3

ST, Sequence Type; SCC*mec*, Staphylococcal cassette chromosome *mec*; MRSA, Methicillin-resistant *S. aureus*; M, Month; D, Day; Y, Year; M, Male; F, Female; SC, Solid cancer; D, Diabetes; C, Cardiopathy; HC, Hematological cancer; L, Lupus; ESRD, End stage renal disease; P, Pneumopathy; NA, Not Applicable; Gen Surg, General Surgery; Int Med, Internal medicine; Hem, Hematology; Emer, Emergence Room; VAN, Vancomycin; PIP, Piperacillin; TAZ, Tazobactam; CEFP, Cefepime; HA, Hospital acquired; HCA, Healthcare associated; OXA, Oxacillin; DAP, Daptomycin; MIC, Minimal inhibitory concentration; PVL, Panton-Valentine leukocidin; ACME, Arginine catabolic mobile element; PFGE, Pulsed field gel electrophoresis

^†^Death as outcome

## Discussion

In this study, we characterized consecutive *S. aureus* isolates from BSI in a teaching hospital in Rio de Janeiro for three years and the emergence of the pandemic CA-MRSA USA300/ST8/SCC*mec*IVa lineage was detected for the first time in this hospital. The patients with USA300 BSI were in wards that were attended by the same health professionals and, therefore, we hypothesized a cross-transmission of these isolates occurred in the hospital. Although other hospitals may not have the same epidemiological situation seen here, these results may reflect a picture of the circulating strains of *S. aureus* that cause BSI in Brazil.

Although the CA-MRSA lineages are classically related to SSTI, they have also been widely implicated in HA-BSI [30, 31]. According to Kourtis et al. [32] CA-MRSA infections provide a reservoir that contributes to the incidence of healthcare-associated diseases and fuels transmission both within and outside healthcare settings. Our group has been carrying out a surveillance of *S. aureus* BSI in this hospital (HUCFF) for years and, although we have described the presence of community *S. aureus* lineages in this hospital since 2009 [8, 10, 33–35] we had not yet seen the presence and spread of USA300/ST8/SCC*mec*IVa. Some Brazilian studies have shown the presence of CA-MRSA isolates in hospitals recovered from different clinical sources, including occasionally USA300/ST8/SCC*mec*IV isolates [36–39]. However, these studies did not characterize the isolates of this lineage accurately. Furthermore, different from what has been described in Latin American countries about the occurrence of the USA300-LV lineage [11], here, the isolates that belonged to USA300/ST8 carried the PVL genes, the SCC*mec* type IVa, as well as the ACME-I cassette, and were characterized as the USA300/ST8 North American lineage. Patients with USA300 BSI presented severe comorbidities and most had a Charlson score ≥ 5 and cancer. Curiously, among the patients with MRSA BSI this pathology was most frequently associated to those presenting USA300 (*p* < 0.05) (data not shown). Although we were not able to demonstrate a time–space relationship among all of them, a close genetic similarity of the isolates leads us to suggest a possible horizontal transmission associated with this clonal lineage. Furthermore, even if all the patients involved did not occupy the same ward at the same time, there would be a possible sharing of unscreened patients, which may be the focus of the outbreak, pointing out the need for constant surveillance of this pandemic clonal lineage within this hospital.

The present study shows a great diversity of MRSA clonal lineages causing BSI which was also observed in previous studies by our group in this teaching hospital [8, 9]. Here, the USA100 lineage is the main MRSA clone (51.4%). These data confirm the replacement of the prior prevalent BEC clone, which now represents only 5.4% of MRSA isolates found. Bride et al. [38], in a study conducted in a hospital in the southeast of Brazil characterized *S. aureus* isolates from various clinical sources and the USA100/ST5 lineage was prevalent among HA-infections. However, in our study, most of the USA100 isolates belonged to ST105 (84.2%), a SLV from ST5. Although this is the first report of this lineage in our hospital, it has already been detected in *S. aureus* isolates from BSI in other Brazilian hospitals [8, 9]. Viana and coworkers recently evaluated MRSA isolates from different hospitals in Rio de Janeiro, between 2014 and 2017 and observed the emergence of this SCC*me*cII/ST105 lineage, which has presented multidrug resistance characteristics and seems to be associated with increased evasion when exposed to monocytic cells [40].

The successful spread of *S. aureus* isolates is commonly attributed to their antimicrobial resistance profile, but virulence may also play a crucial role in colonization and infection [41]. In the present study, the presence of PVL genes was evaluated in all MRSA isolates and only the CA-MRSA lineages related to USA300/ST8 and USA1100/ST30 were positive. Besides, USA300 presented the two virulence determinants investigated, PVL genes and ACME-I. Some authors have reported an association between virulence and mortality among patients with *S. aureus* BSI, indicating that the transmission of a virulent pathogen to an already debilitated patient can worsen his clinical condition [7]. However, more studies are needed to better understand the relevance of virulence determinants in *S. aureus* bloodstream infections.

Among Latin American countries, the MRSA isolates accounted for almost half of the *S. aureus* BSI described over the last few years [1, 11]. In Brazil, the MRSA isolation rates vary according to the region. For example, a study conducted at a tertiary oncology care center in Rio de Janeiro detected 26.3% of methicillin resistance among *S. aureus* isolates recovered from BSI [42]. On the other hand, Primo and coworkers [4] showed a MRSA isolation rate of 58.3% among *S. aureus* BSI in a teaching hospital located in the central-west region of Brazil. The MRSA isolation rate in HUCFF in Rio de Janeiro has remained around 30% in BSI caused by *S. aureus*, since 2011, as previously described by our group [10], similarly to the findings of the present study, which reinforces the need for constant surveillance directed towards this pathogen.

A structured management in diagnosis and treatment of *S. aureus* BSI, which differs accordingly to methicillin-resistance, is crucial for an optimal outcome [43]. Vancomycin is the first-line treatment for MRSA BSI worldwide [5], although non-susceptible vancomycin isolates have been already reported [21, 44]. In the present study, no hVISA/VISA (Vancomycin-intermediate *S. aureus*) or VRSA (vancomycin-resistant *S. aureus*) isolates were detected. However, 58.5% of the isolates presented a high vancomycin MIC, and 76% (28/37) of them were MRSA isolates. The clinical efficacy of this antimicrobial can be critically affected by a MIC = 2 mg/L [21]. Thus, daptomycin has become an important alternative to treat MRSA BSI [45]. However, 13.5% of MRSA isolates in the present study were characterized as non-susceptible to daptomycin. Although daptomycin resistance among *S. aureus* remains rare worldwide (< 0.1%) [1], Silva et al. [46] also found isolates non-susceptible to daptomycin (4.7%) in a Brazilian study involving 128 clinical *S. aureus* isolates. We have already described BSI caused by non-susceptible daptomycin *S. aureus* isolates (MIC of 2 and 4 mg/L) among MRSA-VISA isolates in the hospital of the present study [2]. This data is of great concern because of its impact on the patient’s outcome.

In a previous study, da Silva et al. suggested that comorbidities can contribute to a greater susceptibility for acquiring bacterial infections, such as MRSA BSI [47], in elderly patients. We also verified that MRSA BSI was associated with older patients (*p* = 0.03). In fact, in our study most patients (101/113; 89.4%) presented at least one comorbidity, which may indicate a close relationship with age. Although the global prevalence of MRSA BSI in patients with cancer is relatively low [48], in this study this pathogen was detected in 46% of the patients under study. Even though this study demonstrated that elderly and cancer patients were the most affected, we concluded that the high mortality rates found may not be due to MRSA infection, but to the severe health condition of the patients evaluated.

Some limitations of this study include a lack of national epidemiological data on *S. aureus* BSI, which could make comparative analyses in relation to clinical and microbiological aspects related to this type of infection difficult. Furthermore, we only characterized BSI *S. aureus* isolates, and we did not assess the colonized patients to confirm the hospital spread of USA300/ST8. Although we found *S aureus* isolates from other lineages disseminated in the hospital that seemed to be endemic, such as the USA100 lineage, the unusual presence of the pandemic and hypervirulent USA300/ST8 clone must be highlighted. This is because of its ability to establish itself in hospital settings as has been well described in North American studies; highlighting the fact that this lineage could also become endemic in Brazilian hospitals.

## Conclusions

The emergence of MRSA isolates of the USA300/ST8/SCC*mec*IVa lineage was reported in the Brazilian teaching hospital under study. This possibly occurred due to an in-hospital spread among patients with BSI. Therefore, we conclude that this pandemic and hypervirulent lineage may already be circulating among Brazilian hospitals and could be associated to future hospital outbreaks.

## Data Availability

The data sets generated and analyzed during the current study, such as the PFGE and MLST are not publicly available as there is no public database to deposit PFGE results and no new ST was found in the present study. However, these data are available from the corresponding author on request.

## Abbreviations

BSI: Bloodstream infections
SSTI: Skin and soft tissue infection
HA and CA: Hospital and community-associated
MSSA: Methicillin-susceptible *S. aureus*
MRSA: Methicillin-resistant *S. aureus*
BEC: Brazilian endemic clone
CA-MRSA: Community-associated MRSA
BMD: Broth microdilution
MIC: Minimum inhibitory concentration
CAMHB: Cation-adjusted Muller-Hinton broth
PVL: Panton-Valentine leucocidin
ACME: Arginine Catabolic Mobile Element
SCC*mec*: Staphylococcal cassette chromosome *mec*
PFGE: Pulsed-field gel electrophoresis
MLST: Multilocus Sequence Type
HUCFF: Hospital Universitário Clementino Fraga Filho
ATCC: American type culture collection
LV: Latin American variant
TMP/STX: Trimethoprim/sulfamethoxazole
CLSI: Clinical and Laboratory Standards Institute VISA: vancomycin-intermediate *S. aureus*
hVISA: Heteroresistant vancomycin-intermediate *S. aureus*
CFU: Colony-forming unit
BHI: Brain heart infusion
SLV: Single-locus variant
PCR: Polymerase chain reaction
